# Two novel heterozygous variants in *ATP1A3* cause movement disorders

**DOI:** 10.1038/s41439-022-00184-y

**Published:** 2022-02-18

**Authors:** Shogo Furukawa, Sachiko Miyamoto, Shinobu Fukumura, Kazuo Kubota, Toshiaki Taga, Mitsuko Nakashima, Hirotomo Saitsu

**Affiliations:** 1 Department of Biochemistry, Hamamatsu University School of Medicine, Hamamatsu, Japan; 2 Department of Pediatrics, Sapporo Medical University School of Medicine, Sapporo, Japan; 3 Department of Pediatrics, Gifu University Graduate School of Medicine, Gifu, Japan; 4 Division of Clinical Genetics, Gifu University Hospital, Gifu, Japan; 5 Department of Pediatrics, Nagahama City Hospital, Shiga, Japan

**Keywords:** Disease genetics, Epilepsy, Next-generation sequencing, RNA splicing

## Abstract

Variants in *ATP1A3* cause neuropsychiatric disorders, especially those characterized by movement disorders. In this study, we performed whole exome sequencing for two patients with movement disorders and identified two novel heterozygous *ATP1A3* variants, a missense c.2408G>A variant and an indel c.2672_2688+10delinsCAG variant. The unique indel variant occurred at the exon-intron boundary at the 3′ end of exon 19, and mRNA analysis revealed that this variant caused in-frame indel alteration at the Ser891_Trp896 residue.

The Na^+^/K^+^-ATPase transmembrane ion pump is mainly composed of two subunits, a large catalytic subunit (alpha) and a smaller glycoprotein subunit (beta). In humans, Na^+^/K^+^-ATPase has four different α-subunit isoforms, especially the α_2_-isoform and α_3_-isoform (encoded *ATP1A2* and *ATP1A3*), which are mainly expressed in the brain^1^. To date, only these two isoforms have been shown to be related to neurological diseases in humans^2,3^. Heterozygous variants in *ATP1A3* cause rapid-onset dystonia parkinsonism (RDP, OMIM # 128235)^2^, alternating hemiplegia of childhood (AHC, OMIM # 614820)^4^, cerebellar ataxia, areflexia, pes cavus, optic atrophy, sensorineural hearing loss (CAPOS) syndrome (OMIM # 601338), and polymicrogyria^5,6^. Here, we report two novel variants in *ATP1A3* (NM_152296.5) found in patients with movement disorders.

The first patient (patient 1) is a 1-year-old Japanese girl born as a third child of nonconsanguineous healthy parents without asphyxia. Signs of developmental delay were recognized at 4 months with unstable head control. Neurological examination at 5 months revealed poor head control and hypotonia. At 7 months, she started to show foot clonus, hyperreflexia of the patellar tendon and the Achilles tendon, but there was an absence of alternating paralysis, seizures and involuntary movements. Ophthalmological examination at 5 months indicated loss of eye pursuit and normal light reflex but no abnormal eye movements. Biochemical analysis and electroencephalogram (EEG) examination were normal. Brain magnetic resonance imaging (MRI) at one year showed hypoplasia of the brainstem.

The second patient (patient 2) is a 14-year-old Japanese boy who was born as the second child without abnormal perinatal history. His mother and maternal grandfather also developed mild adult onset dystonia of the upper extremities at approximately 40 and 55 years of age, respectively. At 1 year and 9 months, he suddenly developed throwing head back, turning eyes upward, and weakness of the upper limbs lasting several tens of minutes. At 5 years, he began to show gradual, paroxysmal dystonic attacks in his left hand. His muscle stiffness was noticeable outside of the attacks and worsened when he was nervous since 12 years of age. Brain MRI, spinal fluid test, somatosensory evoked potentials, and short-term EEG were normal. Long-term EEG and electromyogram at 12 years showed sudden onset of left-hand dystonia with stress and caffeine load without EEG response.

This study was approved by the Institutional Review Board Committee at Hamamatsu University School of Medicine. After receiving written informed consent, we performed whole exome sequencing (WES) as described previously^7,8^. WES detected two novel *ATP1A3* variants, a missense variant (NM_152296.5:c.2408G>A, p.(Gly803Asp)) and an indel variant (NM_152296.5:c.2672_2688+10delinsCAG), in patients 1 and 2, respectively (Table 1). These variants were not observed in the Genome Aggregation Database v3.1.1 (accessed Aug 2021), 8.3KJPN Allele Frequency Panel (https://jmorp.megabank.tohoku.ac.jp/) or our in-house 218 control exome data. Sanger sequencing revealed that the c.2408G>A variant occurred *de novo* (Fig. 1A), and multiple in silico prediction tools predicted it to be deleterious. Although the c.2408G>A variant has been registered in ClinVar as a pathogenic variant (RCV000525007), detailed clinical features have not been reported. The c.2672_2688+10delinsCAG variant was inherited from his affected mother and found in his elder brother without symptoms (Fig. 1B). The c.2672_2688+10delinsCAG variant consists of a 27-bp deletion spanning a part of exon 19 (17 bp) and intron 19 (10 bp) and a 3-bp (CAG) insertion (Fig. 1C), suggesting that it can cause abnormal splicing. We performed reverse transcription polymerase chain reaction (RT–PCR) using total RNA extracted from peripheral leukocytes derived from patient 2 and a healthy control as previously described^7^. Two different-sized products were amplified in the patient 2 sample (Fig. 1D). Sequencing analysis of cloned PCR products revealed 38-bp intron retention of intron 19 along with 17-bp deletion and 3-bp insertion, leading to in-frame alteration, p. Ser891_Trp896delinsThrAlaGlyCysCysValSerAlaHisArgLysIleProGly: Six amino acid (from Ser891 to Trp896) residues were replaced by another 14 amino acids in the ATP1A3 protein (Fig. 1E). Based on the American College of Medical Genetics and Genomics standards and guidelines, the c.2408G>A and c.2672_2688+10delinsCAG variants were classified as pathogenic and likely pathogenic, respectively (Table 1)^9^.

**Table 1:** Summary of the clinical and genetic findings in the patients.

	Patient 1	Patient 2
Age	1y0m	14 y
Onset	0y4m	1y9m
Sex	Female	Male
Dystonia	−	+
Hemiplegia/Quadriplegia	−	−
Epilepsy	−	−
Abnormal eye movement	−	+
Developmental delay	+	−
*ATP1A3* variants		
cDNA change	c.2408G>A	c.2672_2688+10delinsCAG
Amino acid change	p.(Gly803Asp)	p.Ser891_Trp896delinsThrAlaGlyCysCysValSerAlaHisArgLysIleProGly
SIFT	0	N/A
PolyPhen-2	0.973	N/A
CADD	27.6	N/A
M-CAP	0.962096	N/A
ACMG guideline	Pathogenic	Likely pathogenic
	PS2, PM1, PM2, PP3, PP5	PS3, PM2, PM4

Variat description based on NM_152296.5.

*PolyPhen-2* Polymorphism Phenotyping v2, *CADD* Combined Annotation Dependent Depletion, *SIFT* Sorting Intolerant From Tolerant, *M-CAP* Mendelian Clinically Applicable Pathogenicity, *ACMG* American College of Medical Genetics and Genomics.

**Fig. 1:**
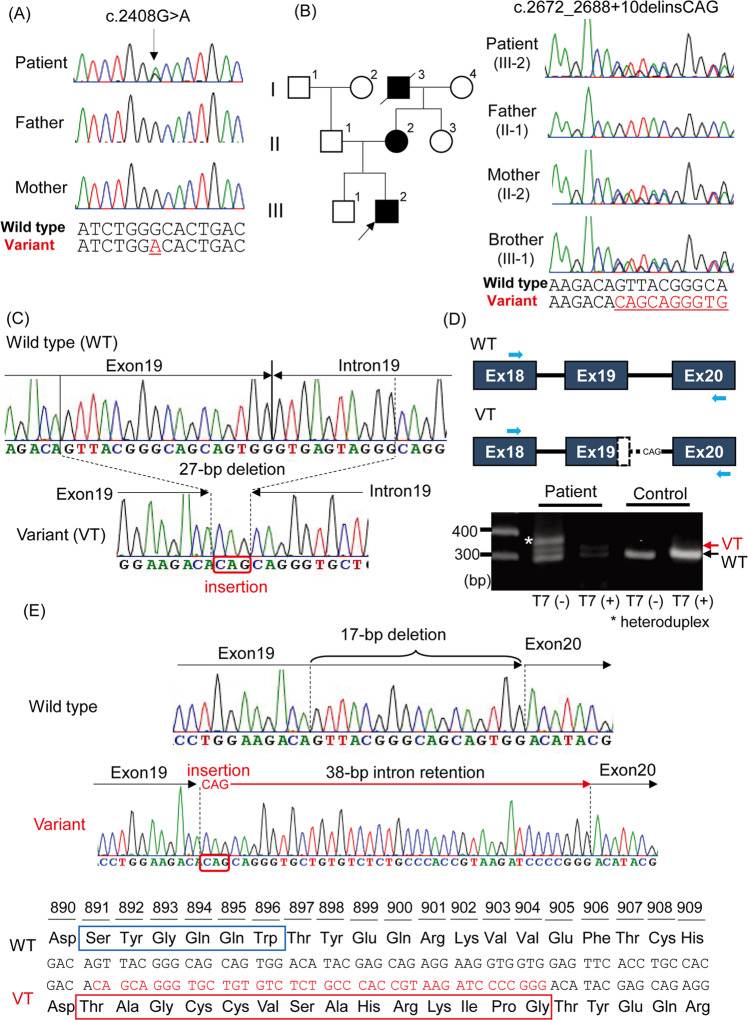
Electropherograms of two family samples. **A** Sanger sequencing reveals that the c.2408G>A variant (arrow) occurred de novo. **B** Family pedigree and electropherogram results of family samples. Sanger sequencing denotes that the c.2672_2688+10delinsCAG variant is inherited from patient 2’s affected mother. **C** Electropherograms of wild-type (top) and variant (bottom) PCR clones of the proband. A 27-bp deletion (dashed lines) and 3-bp (CAG) insertion (red square) occurred at the exon 19 – intron 19 boundary in the variant allele. **D** Schematic representation of the ATP1A3 gene structure and electrophoresis of RT–PCR analysis using cDNA derived from the patient and a control. Black boxes, lines and light blue arrows denote the coding exons, introns and RT–PCR primers, respectively. The patient showed two bands of different sizes (black and red arrows). The largest sized band (white asterisk) found in the patient was digested with T7 endonuclease I, indicating heteroduplexes. WT, wild-type, VT, variant. (**E**) cDNA sequences (upper) and amino acid sequences (lower) of WT and variant. Electropherograms of the variant show a 17-bp deletion (dashed line) at the 3′ end of exon 19 and a 3-bp (CAG) insertion and 38-bp intron retention of the 5’ end of intron 19 (red arrow). This indel variant caused in-frame amino acid alteration, and 6 amino acid residues of WT (from Ser891 to Trp896, blue box) were replaced with another 14 amino acids (red box).

Patients with deleterious *ATP1A3* variants shared similar clinical phenotypes, but there were some differences in patients with AHC and RDP. When comparing RDP with AHC, patients with AHC often have paroxysmal episodes and develop symptoms at a younger age (almost < 18 months)^2,3^. Patient 1 showed early onset developmental delay and hypotonia, whereas she also showed atypical findings, including early-onset pyramidal signs and brainstem hypoplasia. Recent studies suggested that AHC-related variants clustered at the transmembrane regions and functional domains, and variants located in exons 8, 14, 17, and 18 tend to be associated with more severe phenotypes^10,11^. Patient 1 had the c.2408G>A, p.(Gly803Asp) variant located at exon 17 in the transmembrane region, consistent with previous studies.

Meanwhile, patient 2 had the c.2672_2688+10delinsCAG, p. Ser891_Trp896delinsThrAlaGlyCysCysValSerAlaHisArgLysIleProGly variant located at the extracellular region of ATP1A3. Recent studies have indicated that both missense and in-frame variants in this extracellular region cause various types of neurological disorders, including AHC, CAPOS, and polymicrogyria^6,12^. Patient 2 showed an intermediate phenotype between RDP and AHC due to the relatively late onset and the presence of episodic symptoms. His mother and maternal grandfather showed a typical and mild clinical course of RDP, including late onset one-sided limb dystonia that worsened when they were nervous and parkinsonism that was observed in the grandfather. Meanwhile, his elder brother, two years older than patient 2, has not yet developed any neurological symptoms. These findings suggested that this splicing variant might cause RDP with variable severity. The genotype-phenotype correlation and penetrance of *ATP1A3*-related disorders are variable^13,14^, and some familial cases have a broad range of severities^15,16^. However, there are only a few reports showing phenotypic variability within families with *ATP1A3* variants. Here, we precisely described the onset time and specific symptoms or severity for each individual in the family, clearly presenting an example of intrafamilial variability of ATP1A3-related disorders.

## Data Availability

The relevant data from this Data Report are hosted at the Human Genome Variation Database at 10.6084/m9.figshare.hgv.3128, 10.6084/m9.figshare.hgv.3131.
